# Chemical p38 MAP kinase inhibition constrains tissue inflammation and improves antibiotic activity in *Mycobacterium tuberculosis*-infected mice

**DOI:** 10.1038/s41598-020-70184-x

**Published:** 2020-08-12

**Authors:** Christoph Hölscher, Jessica Gräb, Alexandra Hölscher, Annie Linnea Müller, Stephan C. Schäfer, Jan Rybniker

**Affiliations:** 1 Division of Infection Immunology, Research Center Borstel, Parkallee 30, 23845 Borstel, Germany; 2 German Center for Infection Research (DZIF), Partner Site Borstel, 23845 Borstel, Germany; 3 Department I of Internal Medicine, Division of Infectious Diseases, University of Cologne, Robert-Koch Strasse 21, 50931 Cologne, Germany; 4 Center for Molecular Medicine Cologne, University of Cologne, 50931 Cologne, Germany; 5 German Center for Infection Research (DZIF), Partner Site Bonn-Cologne, 50937 Cologne, Germany; 6 Institute for Pathology, University of Cologne, Kerpener Strasse 62, 50937 Cologne, Germany; 7 Institut für Pathologie Im Medizin Campus Bodensee, Röntgenstrasse 2, 88048 Friedrichshafen, Germany

**Keywords:** Tuberculosis, Antimicrobial responses, Immune cell death, Infectious diseases, Inflammation, Infection

## Abstract

Host-modulating therapies have become an important focus in the development of novel concepts for improved management of tuberculosis (TB). Previous in vitro studies revealed that the p38 MAP kinase signaling pathway coordinates several inflammatory and stress responses in *Mycobacterium tuberculosis* (*Mtb*)-infected host cells. Here we extend these findings and show that in vivo treatment of *Mtb*-infected C57BL/6 mice with doramapimod, a p38 MAP-kinase inhibitor, results in reduced inflammation, granuloma formation and lung pathology. Moreover, doramapimod, together with standard antibiotic treatment, significantly reduced lung and spleen mycobacterial loads compared to antibiotic treatment alone. Our in vivo data suggest the opportunity to repurpose p38 MAPK inhibitors for adjunct host directed therapies. We also provide first data on safety of p38 MAPK inhibition which is of relevance for future application of these substances in inflammatory diseases and concomitant TB.

## Introduction

*Mycobacterium tuberculosis* (*Mtb*), the causative agent of tuberculosis (TB), is the major killer among infectious agents which led to 1.5 million deaths in 2018^1^. Treatment of TB requires combinations of antibiotics for several months, a strategy which becomes increasingly complicated in times of rising numbers of multi-drug resistant *Mtb*-isolates^1^. Adjunctive host directed therapy (HDT) might improve and accelerate treatment by modifying host pathways targeted by *Mtb*^2–6^. In HDT, one promising approach is to manipulate signaling pathways that contribute to immunopathology by causing hyperinflammation, necrosis and tissue damage. We have recently shown that genetic or chemical inhibition of p38 mitogen-activated protein kinase (MAPK) in vitro results in abrogation of *Mtb*-induced host cell death and decreased release of inflammatory alarmins such as High-Mobility-Group-Protein B1 (HMGB1) ex vivo^7^. p38 MAPK is a validated target in autoimmune and inflammatory diseases and several small molecule inhibitors have been tested in clinical trials mainly against rheumatoid arthritis, psoriasis and Crohn’s disease^8^. This provides an opportunity to repurpose these substances with excellent safety profiles as adjunct therapeutics in combination with anti-mycobacterial drugs against active TB.

As a serine/threonine protein kinase, p38 MAPK plays an important role in orchestrating the immune response of the host by coordinating the release of proinflammatory cytokines such as interleukin (IL-) 1-beta (β) or tumor necrosis factor (TNF) upon infection^8^. Clinical application of p38 MAPK inhibitors theoretically poses a risk for disease exacerbation and reactivation of TB in latently infected patients (LTBI) as seen for inhibitors of TNF^9^.

In continuation of our previous in vitro findings we consequently sought to determine the potential of p38 MAPK inhibition as an adjuvant treatment for TB and to determine the risk for exacerbation of the disease during monotherapy. To this end, we here analyzed the outcome of experimental TB in vivo under p38 MAPK inhibition with and without antibiotic treatment during acute and chronic infection of C57BL/6 mice.

## Results

### Doramapimod treatment reduces tissue inflammation in the C57BL/6 mouse model of acute *Mtb* infection

**Figure 1:**
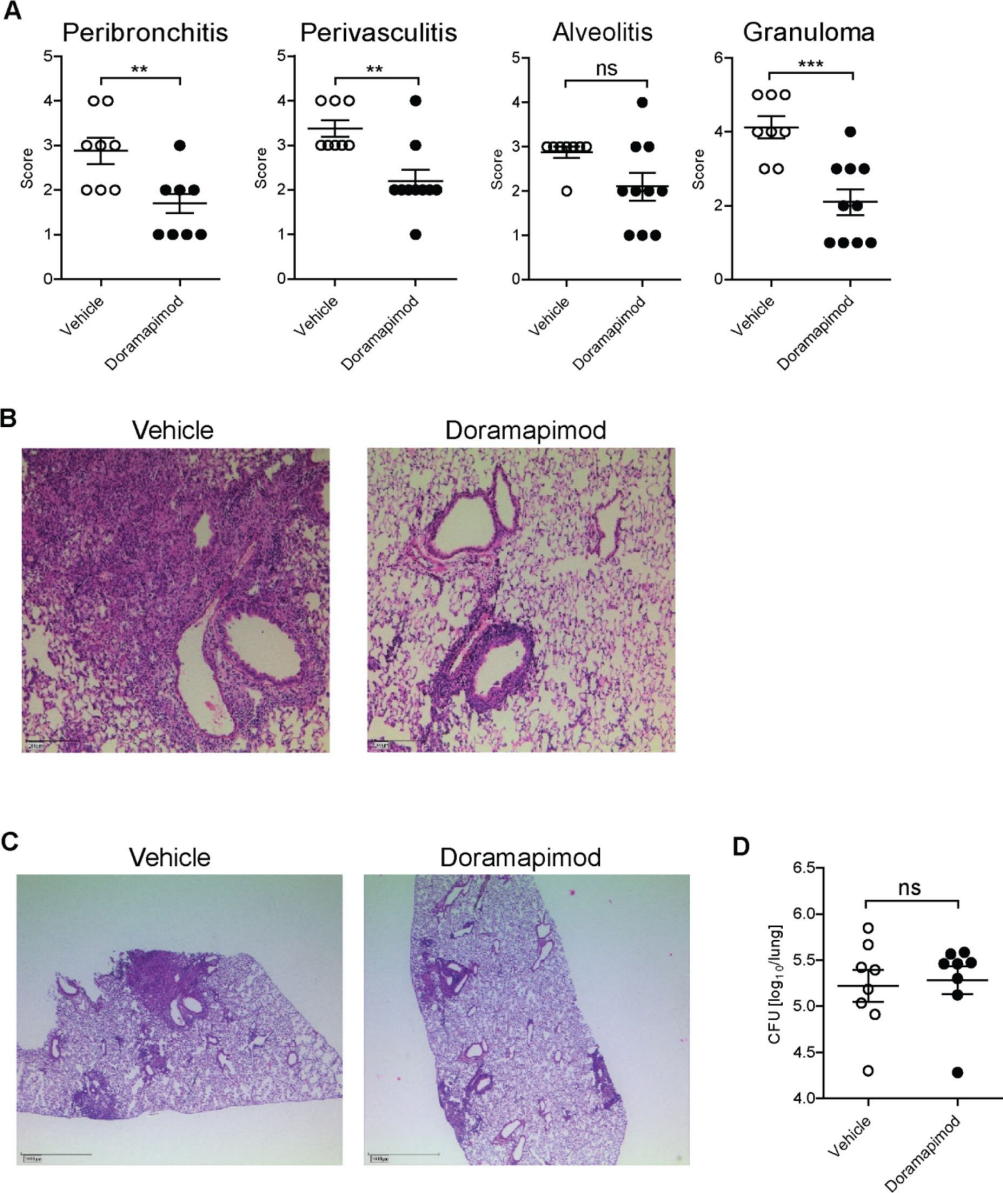
Treatment of mice with doramapimod reduces histopathology in an acute model of Mtb infection. C57BL/6 mice infected with 100 CFU Mtb received doramapimod (30 mg/kg q.d.; oral gavage), while control mice received PEG400 for 28 days. Mice were sacrificed and the histopathological score (**A, B, C**) and the CFU of the lung (**D**) was determined. Data from eight mice per group are shown in (**A**) and (**D**). Representative images of hematoxylin and eosin stained lung lobes are shown in (**B**) and (**C**). Results are expressed as mean ± SEM and experiments were analyzed using unpaired *t* test (ns, not significant; **p ≤ 0.01; ***p ≤ 0.001).

Doramapimod is a well-defined orally bioavailable p38 MAPK inhibitor with confirmed activity in both humans and mice^10^. One day after aerosol infection with *Mtb* H37Rv we started treatment of mice with 30 mg/kg doramapimod or vehicle. After 28 days of treatment, mice were sacrificed and evaluated for histopathological changes and bacterial load in the lungs. In doramapimod-treated mice, lung inflammatory scores such as peribronchitis and perivasculitis were significantly reduced (Fig. 1a,b) and alveolitis was lowered by trend (Fig. 1a). In addition, doramapimod treatment significantly reduced the number of granumlomas (occupied area of the lung section) in infected mice (Fig. 1a,c). Interestingly, a similar bacterial load was found in lungs of doramapimod- and vehicle-treated mice (Fig. 1d). Together, p38 MAPK inhibition during acute experimental TB limits inflammation in the lungs yet does not modulate mycobacterial loads.

### p38 MAPK inhibition impacts *Mtb*-induced inflammatory immune responses in lungs of chronically infected C57BL/6 mice

**Figure 2:**
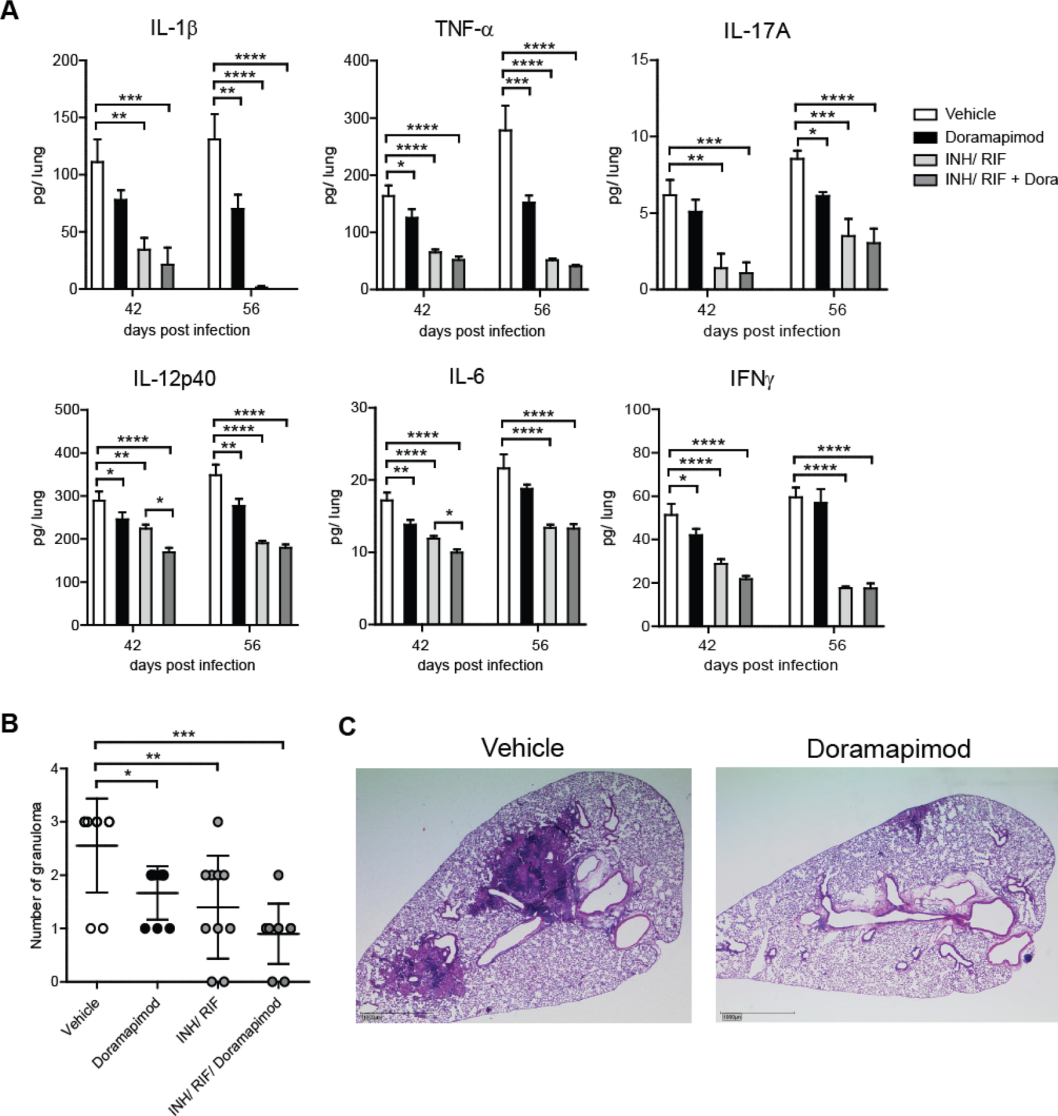
Doramapimod reduces tissue inflammation in chronically infected mice. C57BL/6 mice were infected with 100 CFU Mtb. After 28 days, mice were treated with vehicle (PEG400), doramapimod (30 mg/ kg q.d.), isoniazid (INH; 10 mg/ kg) and rifampicin (RIF; 10 mg/ kg) or INH/RIF and doramapimod. After 42 and 56 days of infection, mice were sacrificed and cytokine levels of lung homogenates were quantified (**A**). The number of granuloma inside the lungs (**B**) was analyzed 56 days post infection. Data derived from 9 to 10 mice are shown in (**A**) and (**B**). Representative images of hematoxylin and eosin-stained lungs are shown in (**C**). Results are expressed as mean ± SEM and experiments in (**A**) and (**B**) were analyzed using one-way ANOVA (*p ≤ 0.05; **p ≤ 0.01; ***p ≤ 0.001; **** < 0.0001). Only significant differences are highlighted.

**Figure 3:**
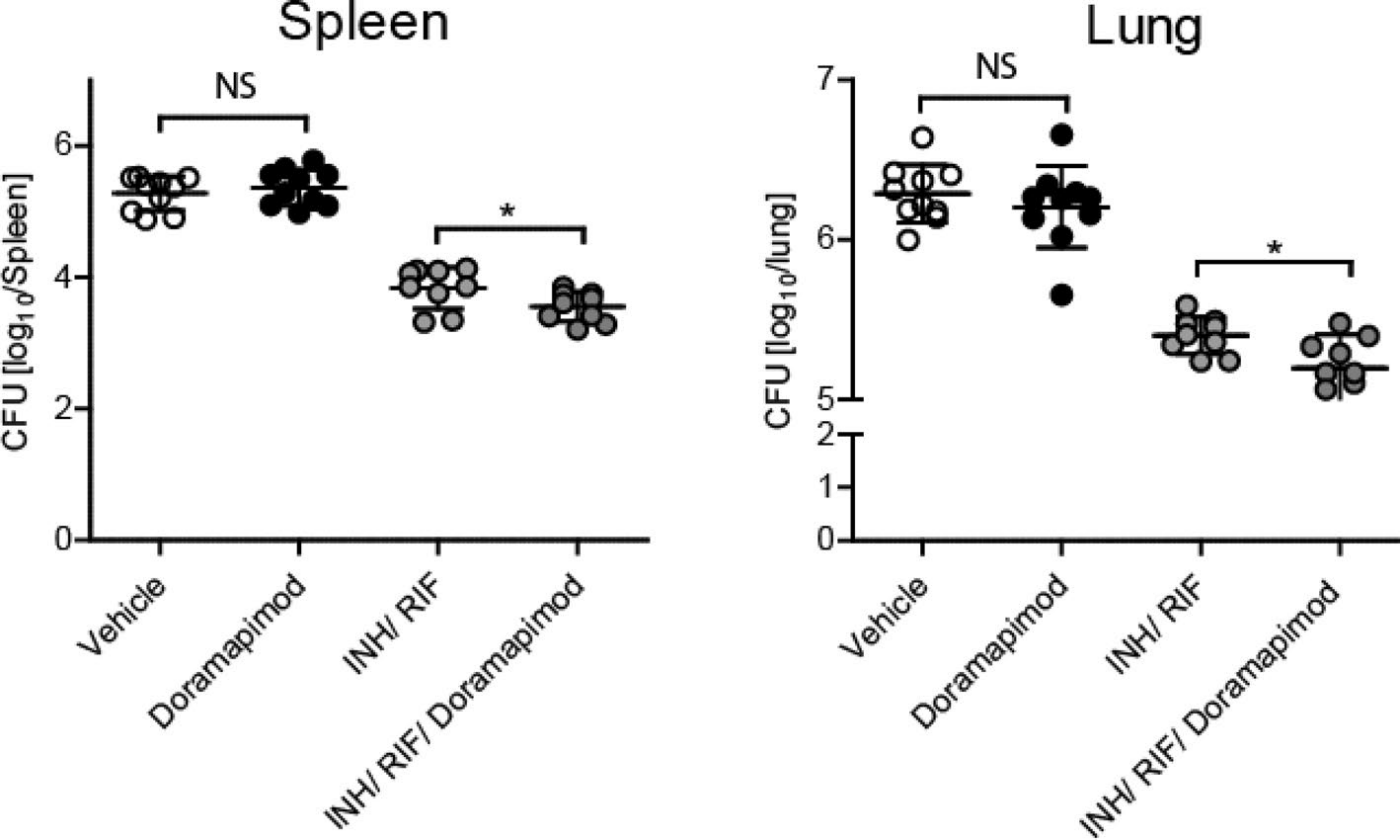
Doramapimod reduces the bacterial load in chronically infected mice. C57BL/6 mice were infected with 100 CFU *Mtb* and 28 days after infection mice were treated with vehicle (PEG400), doramapimod (30 mg/ kg q.d.), isoniazid (INH; 10 mg/ kg) and rifampicin (RIF; 10 mg/ kg) or INH/RIF plus doramapimod. After 56 days of infection, mice were sacrificed and the bacterial load in lungs and spleens was analyzed. Data derived from 9–10 mice are shown and results are expressed as mean ± SEM and analyzed using one-way ANOVA (ns, not significant; *p ≤ 0.05).

To evaluate the impact of p38 MAPK inhibition on the inflammatory immune response, lung infiltration and bacterial loads on an already established disease, we started doramapimod treatment 28 days after aerosol infection of C57BL/6 mice with *Mtb*. Production of pro-inflammatory cytokines in lungs was determined 14 and 28 days after initiation of treatment using cytometric-Bead-Array of lung homogenates. On day 42 of infection, the concentration of IL-6, TNF, IL-12p40 and IFNγ were significantly reduced in doramapimod-treated animals (Fig. 2a; white and black bars). Fourteen days later (day 56), the production of IL-1β, TNF, IL-17A and IL-12p40 remained significantly impaired in treated mice (Fig. 2a; white and black bars). This diminished inflammatory cytokine release in the lungs of doramapimod-treated and *Mtb*-infected mice was accompanied by a strikingly attenuated pulmonary tissue inflammation and granulomatous response (Fig. 2b; white and black circles, and Fig. 2c). Neither doramapimod nor antibiotic treatment had a profound effect on IL-10 production in infected lungs (Supplementary Fig. 1). In contrast to the modulating effect on the inflammatory immune response during chronic *Mtb* infection, doramapimod treatment alone had no impact on bacterial loads in lungs and spleen (Fig. 3; white and black circles). In summary, p38 MAPK inhibition during chronic experimental TB impairs pro-inflammatory immune responses in the lungs but does not affect mycobacterial growth.

### Adjuvant doramapimod treatment positively affects the inflammatory response and reduces the mycobacterial load

We next analyzed the adjuvant effect of doramapimod treatment on the anti-inflammatory and anti-mycobacterial effect of standard antibiotics. To this end, chronically infected C57BL/6 mice received on day 28 of infection doramapimod along with isoniazid and rifampicin and the outcome of experimental TB was compared to mice that received vehicle, doramapimod or antibiotics alone. Overall, treatment with antibiotics resulted in a pronounced decrease of cytokine production in the lungs of *Mtb*-infected animals (Fig. 2a; grey bars). On day 42, the release of IL-6, and IL-12p40 was further reduced by addition of the p38 MAPK inhibitor to the antibiotic treatment (Fig. 2a; grey bars). The initial reduction of cytokine levels in lungs of *Mtb*-infected mice treated with the combination of doramapimod, isoniazid and rifampicin were accompanied by a further reduction of the granulomatous response on day 56 of infection (Fig. 2b; grey circles). Strikingly, this decreased tissue pathology was associated with significantly diminished bacterial loads in lungs and spleen of doramapimod, isoniazid and rifampicin-treated C57BL/6 mice (lung: 5.194 ± 0.201; spleen; 3.548 ± 0.213) when compared to animals that received antibiotics (lung: 5.399 ± 0.119; spleen: 3.837 ± 0.296) alone (Fig. 3; grey circles). Together, p38 MAPK inhibition has an adjuvant impact on the anti-mycobacterial effect of antibiotic treated along with an additive impairment of pro-inflammatory immune responses and tissue pathology.

## Discussion

Targeting signaling pathways which mediate hyperinflammation, necrosis and tissue damage represents a promising approach for adjunctive HDT in management of TB^3^. In this study we followed up on in vitro and ex vivo data in which we were able to link p38 MAPK to inflammation and necrotic cell death in *Mtb* infected host cells^7,11^. We now provide in vivo evidence, that p38 MAPK is a key signaling molecule in *Mtb* pathogenesis. Bacterial infections are well known to activate p38 MAPK either directly by secreted factors and components of the bacterial cell wall or indirectly through the release of pro-inflammatory cytokines like IL-1 or TNF from activated host cells^11^. Therefore, p38 MAPK plays an important role in coordinating the immune response of the host and is often targeted by pathogens to promote virulence and ensure pathogen survival^8^. Histopathological investigation of human biopsies revealed p38 MAPK phosphorylation in macrophages surrounding granulomas in TB patients, indicating that this kinase is engaged and may be a potential target for HDT in TB^12^. Chemical inhibition of p38 MAPK indeed reduced the inflammatory response and granuloma formation in *Mtb* infected C57BL/6 mice (Figs. 1 and 2). Despite significant reduction of cytokines known to be essential for control of the disease in humans and animals, doramapimod treatment had no unfavourable effect on the bacterial load in both acute and chronic infection models of the disease as shown in this work. Similar effects were seen in our ex vivo assays in which doramapimod potently protected infected human macrophages from *Mtb* induced cell death without reducing the intracellular bacterial load^7^. The reason for this may be found in the multiple regulatory effects of this kinase, which not only involve cytokine release but also regulation of autophagy and induction of necrotic host cell death^7,13^. However, our findings stand in contrast to the effects seen with chemical inducers of autophagy such as metformin, an approved drug which is also promoted as a host-directed therapeutic in TB. Induction of autophagy using metformin has little effect on host cell survival but impairs intracellular bacterial growth^14^. Whether p38 MAPK positively or negatively affects autophagy in TB requires further investigations. Nevertheless, it is well-known that *Mtb*-driven activation of the p38 MAPK pathway leads to cyclooxygenase II accumulation, prostaglandin E2 and cyclic AMP release and ultimately matrix metalloproteinase‐1 secretion which are known to play a pivotal role in *Mtb* pathogenesis and tissue destruction^12^. Thus, p38 MAPK inhibition in *Mtb*-infected tissue may generate an immunological microenvironment that is host protective despite the down-regulation of pro-inflammatory cytokines. Interestingly, this environment improves antibiotic activity as shown in our adjunctive treatment experiments with rifampicin and isoniazid (Fig. 3).

Since p38 MAPK is a validated target in autoimmune and inflammatory diseases with several completed or ongoing clinical trials^8^, our findings have important implications for future clinical use of these drugs. We provide first in vivo data on safety of monotherapy with p38 MAPK inhibitors and concomitant *Mtb* infection. It is well known that inhibition of pro-inflammatory cytokines such as TNF in *Mtb*-infected mice results in exacerbation of the disease, increased inflammation, a higher bacterial burden and impaired survival^15^. In contrast, we observed no adverse effects after treatment with doramapimod and subsequent decrease in TNF levels indicating that p38 MAPK inhibition may be less problematic in future applications of these drugs. This requires further confirmation in additional in vivo experiments testing for relapse rates in cured mice. In addition, and most importantly, chemical inhibition of p38 MAPK in *Mtb* infection may represent a highly attractive approach to reduce tissue damage, inflammation and to improve outcome of antibiotic therapy. Further animal studies and clinical trials are warranted.

## Materials and methods

### Mice

C57BL/6 mice were obtained from Charles River (Sulzfeld, Germany). For aerosol infection with *Mtb*, experimental animals were maintained under barrier conditions in individually ventilated cages in the BSL 3 facility at the Research Center Borstel (Borstel, Germany) as described recently^16^. All animal experiments comply with the German Animal Protection Law and were permitted by the Ministry of Energy, Agriculture, the Environment, Nature and Digitalization (Kiel, Germany; permit 113–09/16) after approval by the animal ethics committee of the federal state of Schleswig–Holstein, Germany. We confirm that all experiments were performed in accordance with relevant guidelines and regulations.

### Bacteria and infection

For infection experiments, mice were infected with 100 CFU *Mtb* H37Rv by the aerosol route as published^16^. Before infection of experimental animals, stock solutions of *Mtb* H37Rv were diluted in sterile distilled water and pulmonary infection was performed using an inhalation exposure system (Glas-Col, Terre-Haute, IN, USA). To achieve an infectious dose of approx. 100 CFU/lung, mice were exposed for 40 min to an aerosol generated by nebulizing 6.5 ml of a suspension containing 10^7^ live bacteria. The inoculum size was checked 24 h post infection by determining the bacterial load in the entire lung of infected mice.

### p38 MAPK inhibition and antibiotic treatment

To study the effect of p38 MAPK inhibition on the course of acute *Mtb* infection in C57BL/6 mice, doramapimod (Axon Medchem, Groningen, The Netherlands) was administered by daily oral gavage at a concentration of 30 mg/kg bodyweight (bw) starting at the day one of infection. The corresponding volume of vehicle (polythylenglycol (PEG) 400; Sigma-Aldrich, Taufkirchen, Germany) was administered to control mice. To analyse the adjuvant effect of p38 MAPK inhibition on the treatment with antibiotics, mice were either treated with doramapimod alone, with isoniazid (10 mg/kg bw, Sigma-Aldrich) and rifampicin (10 mg/kg bw, Sigma-Aldrich) or with a combination of doramapimod and antibiotics by daily gavage starting on day 28 of infection.

### Colony enumeration assay

Bacterial loads in lungs and spleens were quantified at different time points after infection with *Mtb* as described earlier^16^. Briefly, organs were removed aseptically, weighed and homogenized. Tenfold serial dilutions of organ homogenates were then plated, incubated at 37 °C and colonies were counted after 21 days.

### Histopathology

For histology, one lung lobe per mouse was fixed in 4% formalin-PBS, set in paraffin blocks, and sectioned (2–3 µm). Histopathological preparations were performed using standard protocols for hematoxylin/eosin staining. Sections were viewed and scored without knowledge of the respective treatment of mice. The histopathological evaluation of sections was performed according to Dormans et al. with minor modifications^17^. Histopathological parameters peribronchiolitis, perivasculitis, alveolitis and granuloma formation were semiquantitatively scored as absent, slight, moderate, marked or strong, noted as 0, 1, 2, 3, 4, and 5, respectively. In this score the frequency as well as the severity of the lesions were incorporated.

Granuloma formation was scored by estimating the occupied area of the lung section.

### Quantification of cytokine production

The concentrations of cytokines in lung homogenates from uninfected and infected mice were determined by a Cytometric-Bead-Array (CBA) (BD Biosciences, Heidelberg, Germany) as described^16^. The quantity of cytokines per lung was calculated based on the ratio of lung to sample weight.

### Statistical analysis

The significance of differences between parameters under different therapies was examined with unpaired* T* tests for Fig. 1 and one-way ANOVA and multiple comparisons for Figs. 2 and 3, where *p* values < 0.05 were considered significant.

## Supplementary information

Supplementary information.
