# Supplementary material for: Chemical p38 MAP kinase inhibition constrains tissue inflammation and improves antibiotic activity in Mycobacterium tuberculosis-infected mice
Source: Sci Rep. 2020 Aug 12;10:13629. doi: 10.1038/s41598-020-70184-x (PMC7423948; doi:10.1038/s41598-020-70184-x)

Supplementary information for:

Chemical p38 MAP kinase inhibition constrains tissue inflammation and improves antibiotic activity in *Mycobacterium tuberculosis*-infected mice

Christoph Hölscher [#] [*] [1, 2], Jessica Gräb[#] [3,4,5], Alexandra Hölscher[1, 2], Annie Linnea Müller [6, 7], Stephan C. Schäfer[6, 7], Jan Rybniker[*] [3,4,5]

[#]equal contribution

[1] Infection Immunology, Research Center Borstel, 23845 Borstel, Germany

[2] German Center for Infection Research (DZIF), Partner Site Borstel, Germany

[3] Department I of Internal Medicine, Division of Infectious Diseases, University of Cologne, 50931 Cologne, Germany

[4] Center for Molecular Medicine Cologne, University of Cologne, 50931Cologne, Germany

[5] German Center for Infection Research (DZIF), Partner Site Bonn-Cologne, Germany

[6] Institute for Pathology, University of Cologne, Kerpener Strasse 62, 50937 Köln

[7] Institut für Pathologie im Medizin Campus Bodensee, Röntgenstrasse 2, 88048 Friedrichshafen

Supplementary Figure 1. p38 MAPK inhibition has no effect on interleukin (IL)-10 expression. C57BL/6 mice chronically infected with Mtb H37Rv (100 CFU/ lung) were treated with vehicle (PEG400), doramapimod (30 mg/ kg q.d), INH (10 mg/ kg) and Rif (10 mg/ kg) or INH/ Rif and doramapimod. 56 days post infection, mice were sacrificed and cytokine levels of lung homogenates were quanitified 42 and 56 days post infection. There was no statistically significant difference.

a

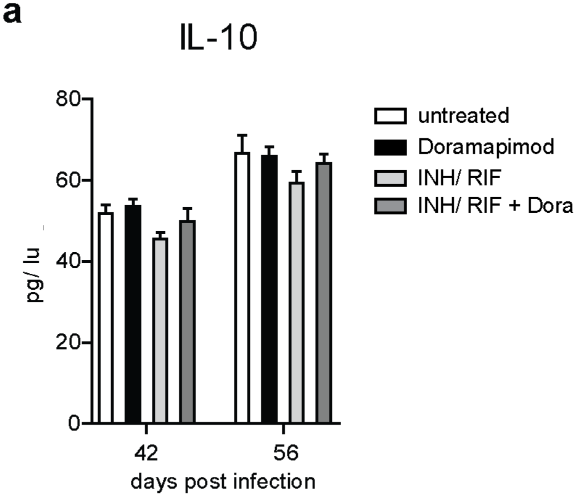

Supplement: Supplementary file 1 — Supplementary information. [file 41598_2020_70184_MOESM1_ESM.pdf]
